# Automating Pitted Red Blood Cell Counts Using Deep Neural Network Analysis: A New Method for Measuring Splenic Function in Sickle Cell Anaemia

**DOI:** 10.3389/fphys.2022.859906

**Published:** 2022-04-05

**Authors:** Amina Nardo-Marino, Thomas H. Braunstein, Jesper Petersen, John N. Brewin, Mathis N. Mottelson, Thomas N. Williams, Jørgen A. L. Kurtzhals, David C. Rees, Andreas Glenthøj

**Affiliations:** ^1^ Centre for Haemoglobinopathies, Department of Haematology, Copenhagen University Hospital, Rigshospitalet, Copenhagen, Denmark; ^2^ Centre for Medical Parasitology, Department of Immunology and Microbiology, University of Copenhagen, Copenhagen, Denmark; ^3^ Department of Haematological Medicine, King’s College Hospital, London, United Kingdom; ^4^ School of Cancer and Pharmaceutical Sciences, King’s College London, London, United Kingdom; ^5^ Core Facility for Integrated Microscopy, Faculty of Health and Medical Sciences, University of Copenhagen, Copenhagen, Denmark; ^6^ KEMRI-Wellcome Trust Research Programme, Kilifi, Kenya; ^7^ Institute of Global Health Innovation, Faculty of Medicine, Department of Surgery and Cancer, Imperial College London, London, United Kingdom; ^8^ Department of Clinical Microbiology, Copenhagen University Hospital, Rigshospitalet, Copenhagen, Denmark

**Keywords:** red blood cell, erythrocyte, sickle cell anaemia, sickle cell disease, splenic function, spleen, PIT count, deep neural network

## Abstract

The spleen plays an important role in the body’s defence against bacterial infections. Measuring splenic function is of interest in multiple conditions, including sickle cell anaemia (SCA), where spleen injury occurs early in life. Unfortunately, there is no direct and simple way of measuring splenic function, and it is rarely assessed in clinical or research settings. Manual counts of pitted red blood cells (RBCs) observed with differential interference contrast (DIC) microscopy is a well-validated surrogate biomarker of splenic function. The method, however, is both user-dependent and laborious. In this study, we propose a new automated workflow for counting pitted RBCs using deep neural network analysis. Secondly, we assess the durability of fixed RBCs for pitted RBC counts over time. We included samples from 48 children with SCA and 10 healthy controls. Cells were fixed in paraformaldehyde and examined using an oil-immersion objective, and microscopy images were recorded with a DIC setup. Manual pitted RBC counts were performed by examining a minimum of 500 RBCs for pits, expressing the proportion of pitted RBCs as a percentage (%PIT). Automated pitted RBC counts were generated by first segmenting DIC images using a Zeiss Intellesis deep learning model, recognising and segmenting cells and pits from background. Subsequently, segmented images were analysed using a small ImageJ macro language script. Selected samples were stored for 24 months, and manual pitted RBC counts performed at various time points. When comparing manual and automated pitted RBC counts, we found the two methods to yield comparable results. Although variability between the measurements increased with higher %PIT, this did not change the diagnosis of asplenia. Furthermore, we found no significant changes in %PIT after storing samples for up to 24 months and under varying temperatures and light exposures. We have shown that automated pitted RBC counts, produced using deep neural network analysis, are comparable to manual counts, and that fixed samples can be stored for long periods of time without affecting the %PIT. Automating pitted RBC counts makes the method less time consuming and results comparable across laboratories.

## Introduction

Despite the spleen playing a key role in the body’s defence against bacterial infections, measuring splenic function remains an area of difficulty. Loss of splenic function is associated with several severe complications, including life-threatening infections with encapsulated bacteria (King and Shumacker, 1952; Barrett-Connor, 1971) and thromboembolic events (Crary and Buchanan, 2009). Measuring splenic function is of clinical interest in multiple conditions, including sickle cell anaemia (SCA) (Brousse et al., 2014). In SCA, spleen damage occurs in early childhood, leading to loss of function within the first years of life (Pearson et al., 1969; Pearson et al., 1985; El Hoss et al., 2019).

The ^99m^Tc sulphur-colloid liver-spleen (LS) scan is often referred to as the “gold standard” for measuring the spleen’s filtration function. The LS scan assesses phagocytic function of both the liver and the spleen, producing a semi-quantitative measurement: the spleen/liver uptake-ratio. However, the vast majority of colloids are taken up by the liver, limiting the diagnostic value pertaining to the spleen (Armas, 1985). An alternative approach is scintigraphy using ^99m^Tc-labelled autologous red blood cells (RBCs). Because labelled RBCs are particularly rigid they get trapped in the spleen, allowing for visualisation of the organ as well as quantitative measurement of splenic uptake (Ehrlich et al., 1982; Gotthardt et al., 2007). These methods are expensive and often not available outside specialised hospital facilities. Furthermore, they require low-dose radiation exposure making them suboptimal for paediatric populations.

Because of the nature of RBC filtration in the spleen, certain RBC changes have been found to act as surrogate biomarkers of splenic filtration. These include nuclear remnants in RBCs, so-called Howell-Jolly bodies (HJBs), and pitted RBCs observed with differential interference contrast (DIC) microscopy. Traditionally, both have been counted manually, HJBs in a May-Grünwald Giemsa-stained blood smear and pitted RBCs in an unstained wet preparation of fresh or fixed whole blood. Results of these splenic biomarkers have previously been compared to results of LS scans and both HJB and pitted RBC counts (PIT counts) correlate well with LS scan results (Pearson et al., 1985; Rogers et al., 2011). However, both methods are user-dependent and time-consuming.

In recent years, a number of techniques have been developed for quantifying HJB-containing RBCs by flow cytometry (Harrod et al., 2007; El Hoss et al., 2018), although to date such methods have been limited to a small number of laboratories. Furthermore, flow cytometry comes with limitations, including the need for rapid analysis following venesection (Harrod et al., 2007).

Pitted RBCs observed using DIC microscopy were first found to predict splenic function more than 50 years ago (Kent et al., 1966; Nathan and Gunn, 1966; Holroyde et al., 1969). Pits are large vacuoles beneath or attached to the RBC membrane (Schnitzer et al., 1971). When studied with DIC microscopy they adopt a 3-dimensional appearance, resembling crater-like indentations or *pits* on the cell surface. The vacuoles contain cellular waste material such as precipitated haemoglobin, degenerated mitochondria and cell-membrane remnants, and form as RBCs age (Nathan and Gunn, 1966; Holroyde and Gardner, 1970). When filtering through a normally functioning spleen, RBCs are groomed by the spleen, removing waste such as pits, before being released back into the circulation. To add confusion, this splenic extraction process is known as *pitting*. In cases where the spleen does not function, pits remain in the circulating RBCs. Thereby, the percentage of RBCs containing pits (the PIT count or %PIT) increases in individuals who have undergone splenectomy (Holroyde and Gardner, 1970; Pearson et al., 1979), or in conditions where there is a loss of splenic function (Casper et al., 1976). In SCA, early studies found that a PIT count of ≥3.5% indicated loss of splenic function (Pearson et al., 1985). More recently, a PIT count of <1.2% was found to predict normal splenic function and a PIT count of >4.5% to predict absent splenic function in SCA (Rogers et al., 2011).

To perform PIT counts, only a small drop of whole blood is needed, making the method ideal for diagnostics in young children. Samples can be analysed either fresh or following fixation, typically using PBS-buffered glutaraldehyde or paraformaldehyde. Previous studies have assessed the durability of fixed RBCs for PIT counts, observing no alterations over periods of up to 3 months (Holroyde et al., 1969; Pearson et al., 1985), and the technique has been found to be reproducible (Pearson et al., 1985).

Although manual PIT counts are a well-validated surrogate marker for splenic function, the method is time-consuming and user dependent. The development of automated methods would be useful for standardising measurements and making results more comparable across laboratories. Furthermore, an automated approach could allow for larger numbers of cells to be counted and, thus, add precision to such analyses. In this study, we propose a new automated workflow for performing PIT counts and assess the durability of fixed RBCs for PIT counts over time.

## Materials and Methods

### Samples

Samples from individuals with SCA and normal controls were included in the study. Individuals with SCA aged 0–16 years were recruited from the paediatric haematology clinic at King’s College Hospital, London. Samples were prepared at King’s College London by mixing 50 μL of EDTA blood with 2 ml 2% PBS-buffered paraformaldehyde. Samples were kept at 4°C and transported to Copenhagen University Hospital for analyses. Normal control samples were randomly selected among surplus blood samples collected into EDTA from healthy adult subjects attending for routine haematology diagnostics at Copenhagen University Hospital.

### Ethical Approval

The National Health Service (NHS) Research Ethics Committee approved this study (ref: 18/LO/1566). For individuals with SCA, written informed consent was obtained from all children aged 16 years prior to sample collection. A parent or guardian provided written informed consent for children under 16 years.

### Microscopy

In preparation for microscopy, samples were gently mixed. When the blood pellet was fully re-suspended, 5 μL of the solution was applied to a glass slide and examined under a #1.5 coverslip after settling for a few minutes.

All microscopy was performed at the Core Facility for Integrated Microscopy, Faculty of Health and Medical Sciences, University of Copenhagen. Samples were examined as a wet-preparation using an oil-immersion objective (×100/1.46) in an inverted Zeiss CellObserver microscope with a DIC setup (condenser NA = 0.55). DIC images of all samples were recorded using a Hamamatsu Orca LT camera.

### Manual PIT Counts

For manual PIT counts, a minimum of 500 consecutive RBCs per sample were examined for the presence of one or more pits (rounded, crater-like depressions on the cell surface). The proportion of pitted RBCs was expressed as %PIT. Manual PIT counts were either performed directly in the microscope eyepieces, or by subsequently counting pitted RBCs on recorded DIC images.

### Developing the Neural Network and Analysis Macro

**FIGURE 1:**
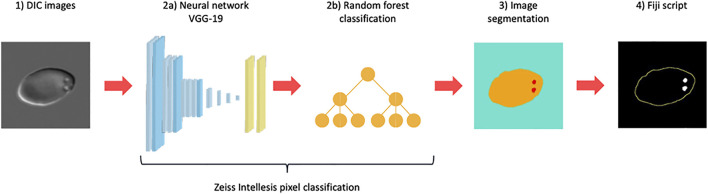
Neural network setup. Differential Interference Contrast (DIC) microscopy images (1) were segmented using a Zeiss Intellesis deep learning model based on the weights used for the VGG19 model (2a) followed by a random forest classifier (2b). The network performs pixel classification and was trained to recognise and segment red blood cells and pits from background (3), creating three channels in total (blue: background, yellow: cell, red: pit). Subsequently, segmented images were analysed and filtered in a postprocessing ImageJ macro script (4), producing an automated pitted red blood cell count.

All image analyses were performed on DIC images taken of wet-preparations prepared in the same way as for manual PIT counts. For all image analyses, DIC images were segmented using Zeiss Intellesis (Carl Zeiss AG) deep learning model (256 features), based on the weights used for the VGG19 model (Simonyan, 2015), provided by TensorFlow, and followed by a random forest classifier. The network performs pixel classification, recognising and segmenting cells and pits from background (three channels in total). The neural network setup is illustrated in Figure 1

**Network 1:** Initially, manual annotation of pits in 99 randomly selected images representing samples from 37 individuals (33 with SCA and 4 normal controls) was performed by three independent observers. The performance of the deep neural network was then compared to the manual pit-annotations and optimised to match the average performance of the manual annotators (data not shown).

**Network 2:** After establishing that a network could successfully be trained to recognise pits and match the average human performance, a new network was trained to recognise both RBCs and pits from background. Several hundred images segmented with Network 2 were reviewed alongside original DIC images, in order to minimise errors and optimise network performance.

For analyses of the segmented images showing three channels (background, cells and pits), we developed a small ImageJ (Rasband, 1997-2018) macro language script. The script splits the three channels, fills in any holes in cell images and separates adjoining cells using a Distance Transform Watershed from MorphoLibJ (Legland et al., 2016). Using the “Analyse Particles” function in ImageJ, only cells of appropriate sizes for RBCs were selected and any cells touching the edge of an image were discarded. Within the regions of interest (ROIs) of selected cells, maxima of the segmented pits were counted, and the summed area of pits was measured. Finally, results were written in Excel-format, using the “Read and Write Excel” plugin (Sinadinos, 2020).

In summary, Network 1 was trained to only recognise pits and used to explore whether a network could recognise pits as reliably as human annotators. Network 2 (the final network) was trained to recognise both RBCs and pits, and segmented images were subsequently analysed and filtered in a postprocessing ImageJ macro script (Figure 1).

### Sample Durability

In order to assess sample durability, three samples were kept refrigerated at 4°C for up to 24 months after sampling: two samples from individuals with SCA and one normal control sample. DIC images were recorded and manual PIT counts performed at varying time intervals on all three samples.

After 24 months of storage, each sample was divided in four, in order to further assess durability at varying temperatures. For each sample, one part remained refrigerated at 4°C, one was incubated at 37°C, one was stored at room temperature in the dark and one at room temperature exposed to daylight (not direct sunlight). After an additional 6 weeks of storage, new DIC images were obtained, and manual PIT counts were performed.

### Statistical Methods

When assessing sample durability, manual PIT counts were compared using multiple comparison ANOVA. Manual PIT counts were compared to automated counts with a paired *t*-test. Significance level was defined as *p* < 0.05. Agreement between manual and automated PIT counts was assessed with a Bland Altman (BA) plot (Martin Bland and Altman, 1986). For the overall population, limits of agreement (LOAs) were deemed acceptable if they were within ±5% of the manual %PIT. For samples with a manual %PIT of <5%, LOAs were acceptable if they were within ±1.5%. Statistical analyses were performed in Stata V16.1 (StataCorp, Timberlake, USA). BA calculations and plots were created with “R” version 3.6.2 (R Development Core Team, 2017) using the blandr and ggplot2 packages.

## Results

### Manual PIT Counts

In order to assess any variability in manual counts, PIT counts for three samples (one normal control and two SCA samples) were repeated three times directly in the microscope eyepieces and three times by examining DIC images on a computer screen, all by the same observer. These three samples were not included in any network validation. Two samples had a PIT count of <5% (one normal control and one SCA sample), with manual counts ranging from 0.4% to 0.8% and 3.9% to 4.6%, respectively. One SCA sample had a high %PIT, with manual counts ranging from 34.1% to 38.6%.

### Network Validation

**FIGURE 2:**
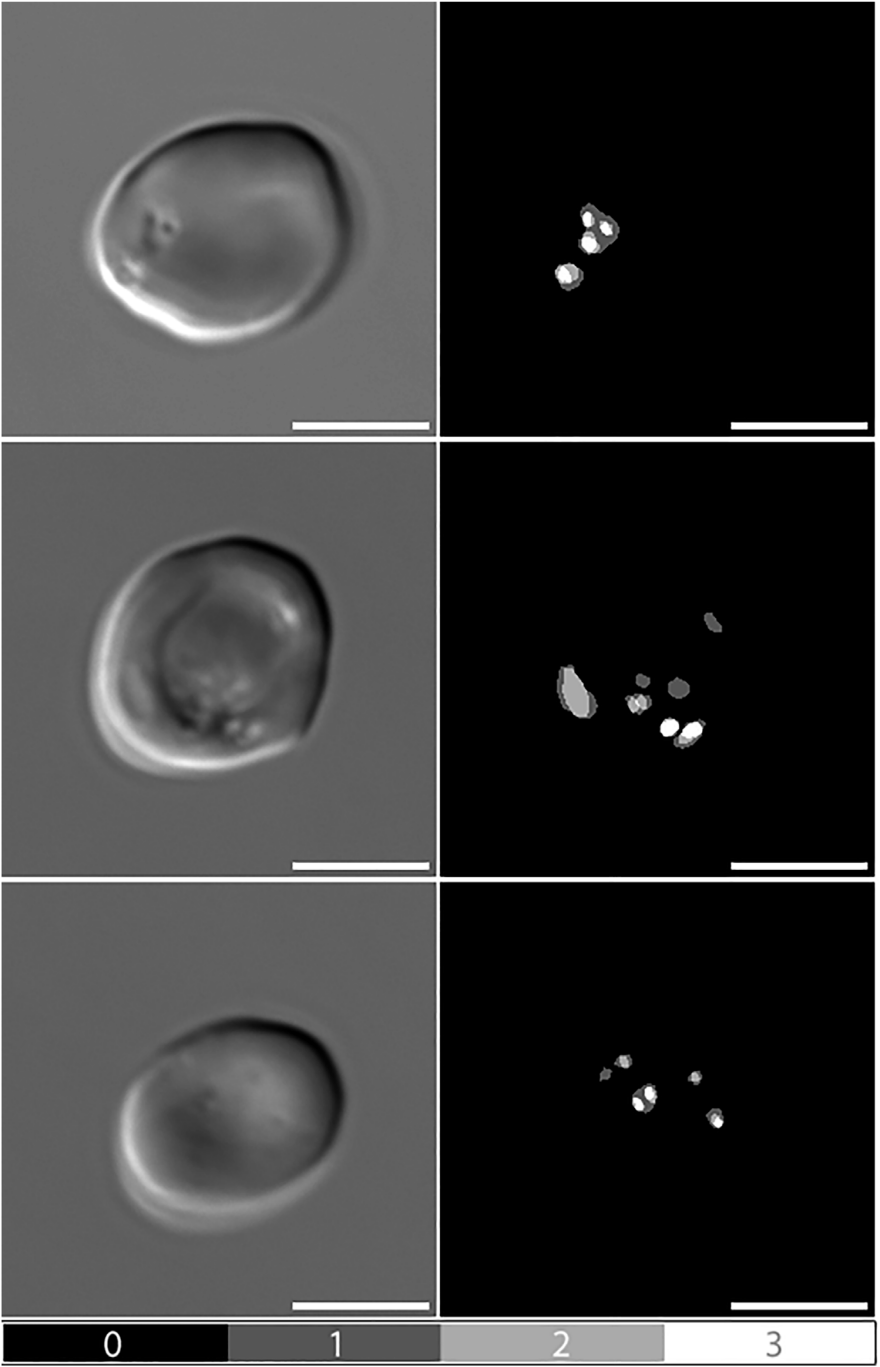
Manual pit-annotations. Differential Interference Contrast (DIC) microscopy images of pitted red blood cells, alongside manual annotations by three independent observers. Dark grey areas indicate annotations made by a single observer (1), light grey indicates consensus between two observers (2), and white indicates consensus between all three observers (3). Scale bars = 5 μm.

When comparing manual pit-annotations to the performance of Network 1, results were very similar. Figure 2 presents examples of manual pit annotations by three independent observers, emphasising differences in the perception and annotation of pits. The three annotators had identified pits in 45.6, 46.1 and 47.9% of 697 RBCs, respectively. In comparison, Network 1 had identified pits in 44.2% of the cells.

**FIGURE 3:**
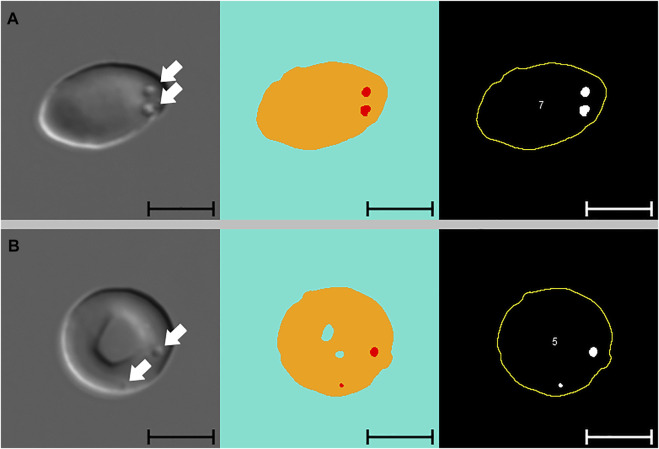
Pitted red blood cells. Differential Interference Contrast (DIC) microscopy images of pitted red blood cells **(A** and **B)**, alongside segmented images and results from subsequent macro analysis (from left to right). Segmented images display three channels, blue: background, yellow: cell, red: pit. White arrows indicate the position of pits. Cell numbers only refer to the numbering of cells reported by the macro. Scale bars = 5 μm.

**FIGURE 4:**
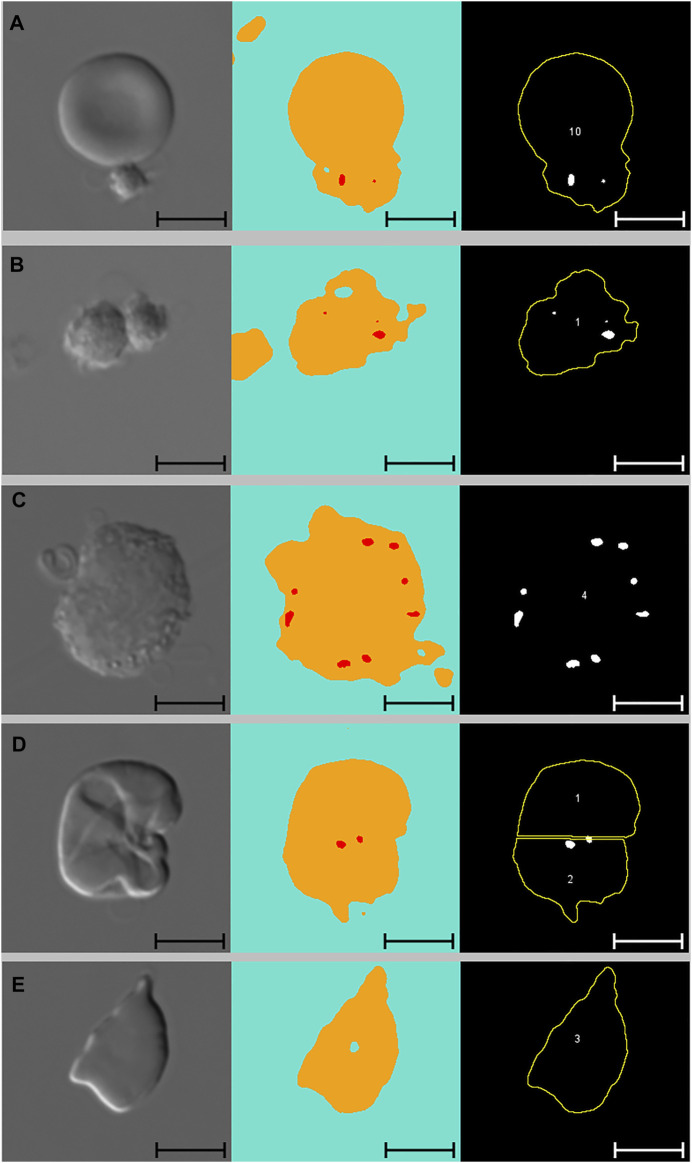
Artefacts. From left to right: DIC microscopy images, alongside segmented images displaying three channels (blue: background, yellow: cell, red: pit), and results from subsequent macro analysis. Scale bars = 5 μm. **(A)** Single platelet and RBC. The RBC has no pits but due to the adjoining platelet, the neural network identifies the cell as pitted. This cell was not excluded from the macro analysis due to normal solidity and size. **(B)** Two adjoining platelets identified by the neural network as one RBC with multiple pits. This “cell” was ultimately excluded from the macro analysis due to low solidity. **(C)** WBC identified by the neural network as a RBC with multiple pits. This cell was ultimately excluded from the macro analysis due to size (no yellow outline). **(D)** Wrinkled RBC from SCA sample identified by the neural network as having multiple pits and subsequently divided into two cells by the analysis macro. Cell no. 2 was ultimately excluded from the analysis due to low solidity. **(E)** RBC from SCA sample. Despite the irregular shape, this cell was not excluded from the analysis as its solidity was normal. *DIC: differential interference contrast, RBC: red blood cell, SCA: sickle cell anaemia, WBC: white blood cell*.

**FIGURE 5:**
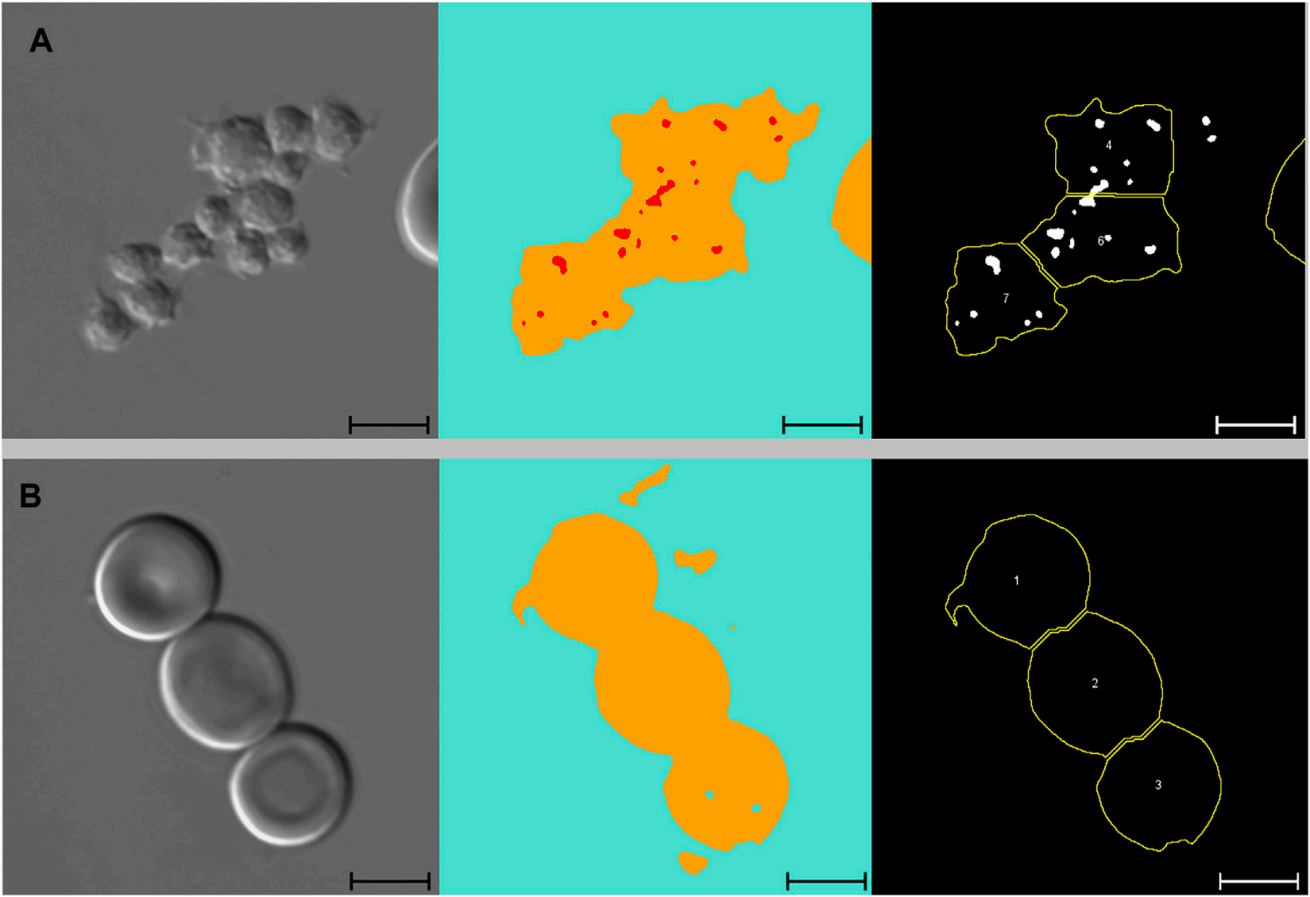
Platelets and adjoining red blood cells. From left to right: DIC microscopy images, alongside segmented images displaying three channels (blue: background, yellow: cell, red: pit), and results from subsequent macro analysis. Scale bars = 5 μm. **(A)** Large platelet-aggregate identified by the neural network as adjoining RBCs with multiple pits. Subsequently divided into multiple “cells” in the macro analysis. All three “cells” were ultimately excluded from the analysis due to low solidity. **(B)** Three adjoining RBCs with no pits correctly identified by the neural network and subsequently divided correctly in the macro analysis. Cell no. 1 was ultimately excluded from the analysis due to low solidity. *DIC, differential interference contrast; RBC, red blood cell*.

Figure 3 presents images of two cells with pits, alongside images segmented with Network 2 and results of the analysis macro. When comparing images segmented with Network 2 to original DIC images, several false-positive events were identified. Examples of such artefacts are illustrated in Figure 4 and Figure 5. The main issues were:

1) Platelets lying on the edge of a RBC were interpreted by the network as being part of the RBC, thereby marking the RBC as having one or more pits (Figure 4A).
2) Platelet-aggregates were interpreted by the network as RBCs with many pits and were not excluded by size in the subsequent macro analysis (Figure 4B and Figure 5A).
3) White blood cells were mostly excluded due to size (Figure 4C), but some smaller cells (presumably lymphocytes) were interpreted by the network as RBCs with many pits.
4) Very wrinkled cells, observed primarily in SCA samples (possibly dehydrated or irreversibly sickled cells), in which it was impossible to distinguish whether there were any true pits. In a manual count such cells would typically be excluded by the observer. In the automated analysis, however, these cells were not excluded and were interpreted by the network as having multiple pits (Figure 4D).

By excluding cells with low solidity (calculated as the area divided by the convex area, showing the object’s irregularity of its boundary), we managed to minimise the number of false positive events caused by platelets (both as aggregates and in relation to RBCs). When filtering by solidity, some normal and sickle cells were inevitably excluded, although not in large numbers. Figure 4E illustrates how a sickle cell, despite its irregular shape, does not get excluded based on solidity. Figure 5B illustrates a normal cell that is excluded due to low solidity (cell number 1), as the small piece of debris attached to the left side of the cell is recognised as being part of the cell.

For normal control samples, the mean difference between manual and automated counts before excluding cells with low solidity was 1.7% (range: 0.6% to 3.3%). After excluding cells with low solidity, the mean difference was 1.0% (0.5% to 1.8%). For the total 58 samples, the mean difference between manual and automated counts before excluding cells with low solidity was 2.0% (−5.9% to 8.7%). After excluding cells with low solidity, the mean difference was 0.6% (−6.7% to 6.3%).

**FIGURE 6:**
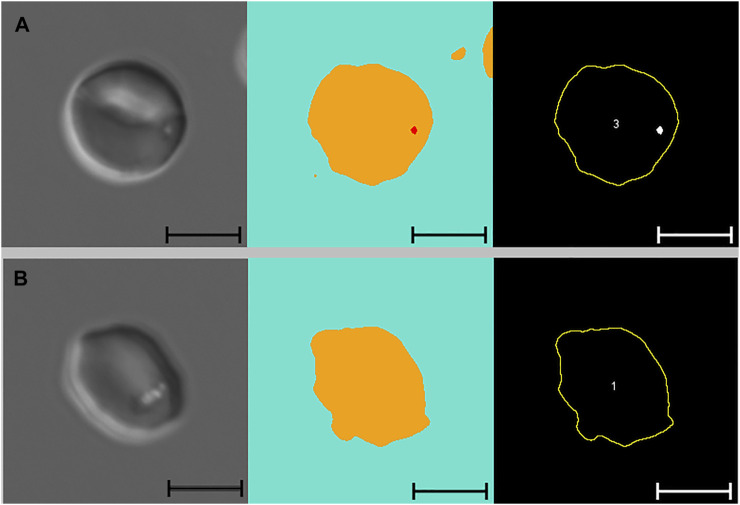
Out of focus images. From left to right: Out of-focus DIC microscopy images of pitted RBCs, alongside segmented images displaying three channels (blue: background, yellow: cell, red: pit), and results from subsequent macro analysis. **(A)** RBC with a single pit correctly identified by the neural network despite the image being slightly out of focus. **(B)** RBC with multiple pits that are not identified by the neural network because the image is too out of focus. *DIC, differential interference contrast; RBC, red blood cell*.

Some false-negative events were also observed, most of which were caused by out of focus images. Figure 6 illustrates how the network failed to identify pits in some, but not all, out of focus images.

### Comparing Automated and Manual Counts

Automated PIT counts performed using Network 2 were compared to manual PIT counts in samples from 58 individuals: 48 individuals with SCA and 10 normal controls. When analysing results from all 58 samples, we found no significant difference in mean %PIT between manual and automated counts (mean ± standard deviation (SD), manual: 30.4% ± 18.4; automated: 31.0% ± 18.8, *p* = 0.1).

**FIGURE 7:**
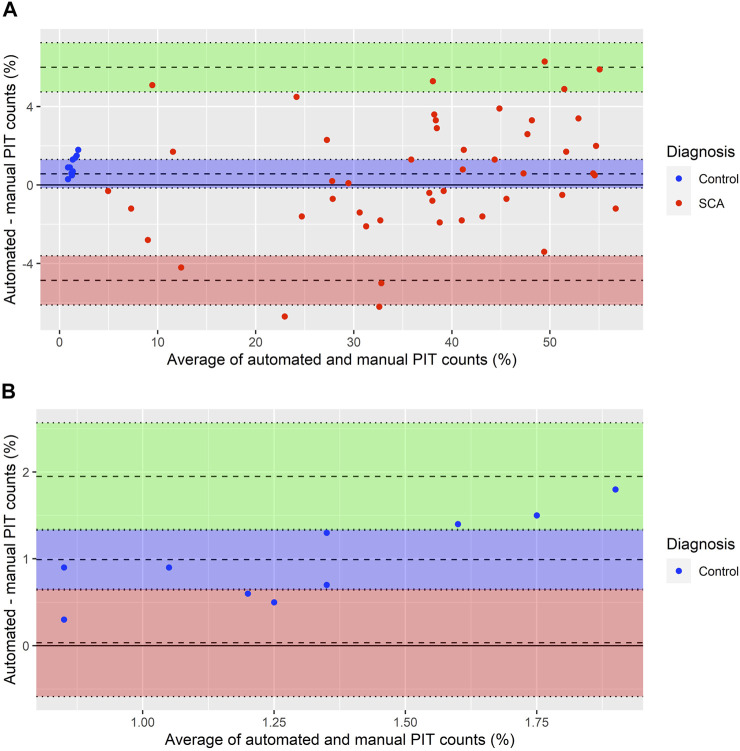
Bland Altman plot comparing manual and automated PIT counts. **(A)** Bland Altman plot of 58 single paired measurements of automated and manual PIT counts. Averages lie between 0.85% and 56.7%. Differences lie between −6.7% and 6.3%. Mean difference between measurements (bias) = 0.57% (95% CI: −0.16% to 1.30%, blue area). The points center around the bias, surrounding it more tightly on the left and spreading out to larger variability on the right. Upper limit of agreement = 6.00% (95% CI: 4.75% to 7.26%, green area). Lower limit of agreement = −4.86% (95% CI: −6.11% to −3.61%, red area). **(B)** Bland Altman plot of automated and manual PIT counts in 10 normal control samples [blue data points in **(A)**]. Averages lie between 0.85% and 1.9%. Differences lie between 0.3% and 1.8%. Mean difference between measurements (bias) = 0.99% (95% CI: 0.64% to 1.34%, blue area). All points are above the bias, suggesting some systematic error with higher values in automated compared to manual counts. Upper limit of agreement = 1.95% (95% CI: 1.33% to 2.57%, green area). Lower limit of agreement = 0.03% (95% CI: −0.57% to 0.65%, red area).

A BA-plot comparing automated and manual counts in all 58 samples is presented in Figure 7A. The plot suggests that the two measurements are in agreement, although with some random relative error: as the %PIT increases, so does the variability of the measurements. The mean difference between measurements (bias) was 0.57% (95% confidence interval (CI): −0.16% to 1.30%). The upper LOA was 6.00% (95% CI: 4.75% to 7.26%) and lower LOA −4.86% (95% CI: −6.11% to −3.61%). Four measurements (6.9%) fell outside the LOAs, all were SCA samples with a %PIT of >20%.

Figure 7B presents a BA-plot for the samples with a %PIT of <5%, all of which were normal controls. For these 10 samples, the bias was 0.99% (95% CI: 0.64% to 1.34%). The upper LOA was 1.95% (95% CI: 1.33% to 2.57%) and lower LOA was 0.03% (95% CI: −0.57% to 0.65%), with no samples falling outside the LOAs. There were signs of some systematic error, with all automated PIT counts being higher than manual counts.

### Number of Pits Per Cell and Pit Size

Additional data generated by Network 2 included the number of pits per pitted cell and the average pit size expressed as a percentage of the total area (100%) of the affected RBC.

The average number of pits per pitted cell ranged from 1.1 to 2.1, with lower numbers in normal controls (mean ± SD: 1.19 ± 0.13) compared to individuals with SCA (1.56 ± 0.22). There was a very strong positive correlation between %PIT and the average number of pits per pitted cell (r = 0.85, *p* < 0.001). Similarly, the maximum number of pits found in one cell ranged from 2 to 12, with lower numbers in normal controls (3.2 ± 0.9) compared to individuals with SCA (8.0 ± 2.1). There was a strong positive correlation between %PIT and the maximum number of pits found in one cell (r = 0.79, *p* < 0.001). Finally, average pit size ranged from 0.21 to 0.76% of the affected cell, with lower numbers in normal controls (0.31% ± 0.07) compared to individuals with SCA (0.57% ± 0.12). There was a strong positive correlation between %PIT and average pit size (r = 0.84, *p* < 0.001).

In samples from the 48 individuals with SCA, we found that the average number of pits per pitted cell (r = 0.36, *p* = 0.01) and average pit size (r = 0.34, *p* = 0.02), as well as %PIT (r = 0.37, *p* = 0.01), all correlated with age.

### Sample Storage

Manual PIT counts were performed on DIC images taken at 1 week, 6 weeks, 5 months, 1 year and 2 years from sampling. Between counts, %PIT for the two SCA samples ranged from 33% to 35.1% and 20.4% to 21.9%, respectively. %PIT for the control sample ranged from 0.6% to 1.2%. Results showed that %PIT did not change significantly over this period (*p* = 0.6).

Subsequently, manual PIT counts were performed on DIC images taken after an additional 6 weeks of storage under varying conditions: refrigerated at 4°C, incubated at 37°C, at room temperature in the dark and at room temperature in daylight. %PIT for the two SCA samples ranged from 31.9% to 34.8% and 21.9% to 23.2%, respectively. %PIT for the control sample ranged from 0.4% to 0.6%. Results showed that %PIT did not differ significantly between storage conditions (*p* = 0.7).

## Discussion

By training a deep neural network to recognise and segment RBCs and pits in DIC microscopy images, we developed a novel automated workflow for counting pitted RBCs. Our findings suggest that automated PIT counts can potentially replace labour-intensive manual counts.

Prior to comparing manual and automated PIT counts, we had repeated manual PIT counts for three selected samples with varying %PIT: one was a normal control sample with a manual PIT count of <1%, one was a SCA sample with a relatively low PIT count of <5%, and one was a SCA sample with a high PIT count of >30%. These repeated counts revealed there to be variability between %PIT for the same sample, even when counted by one observer. For the two samples with a %PIT of <5% the variability was low, whereas the sample with a high %PIT had greater variability. Based on these findings, we decided to accept greater differences between manual and automated PIT counts in samples with high %PIT, compared to samples with low %PIT.

When comparing manual PIT counts to automated counts generated by the neural network we found no significant differences in samples from children with SCA or normal controls. When further evaluating agreement between manual and automated PIT counts in a BA-plot, we found the mean difference between measurements (the bias) to be low, suggesting that the two methods yielded comparable results. The positive bias indicated that automated counts were on average slightly higher than manual counts. There was some random relative error, with increased variability at higher %PIT, a trend similar to what we had identified when repeating manual counts, but there was no evidence of systematic error. Although the upper LOA of 6% was higher than we had aimed for, we did not find this to significantly affect results. All four samples that fell outside the LOAs, as well as the three samples that were within the LOAs but had a variability over the aim of ±5%, had manual PIT counts of >20%. In samples with PIT counts of this magnitude, this variability does not change the diagnosis of functional asplenia.

In contrast, such variability in samples with low %PIT may affect the final diagnosis. In samples with a %PIT of <5%, which included all normal controls, the mean difference between measurements was 0.99%. The BA plot for these samples indicated some systematic error, as all automated PIT counts were higher than manual counts. Furthermore, the upper LOA of 1.8% was higher than our aim of ±1.5%, although just one sample had a variability above 1.5%. Previous studies that compared manual PIT counts to ^99m^Tc sulphur-colloid LS-scan results (Pearson et al., 1985; Rogers et al., 2011), have proposed PIT counts under 3.5 and 1.2%, respectively, to be indicative of normal splenic function in SCA. Despite our automated PIT counts being higher in samples with low %PIT, results for all normal controls remained <3.5% and thus, when following this cut-off value, did not generate a false diagnosis of functional asplenia. Furthermore, all normal control samples had lower %PIT than all SCA samples, regardless of the method. It is possible that normal ranges need to be re-evaluated for automated counts, preferably by comparing these to scintigraphy using ^99m^Tc-labelled autologous RBCs.

The %PIT generated for 99 DIC images by Network 1 (identifying only pits), was very similar to those counted by the three manual annotators. However, when reviewing images segmented with Network 2 (identifying both pits and RBCs), we identified a high number of false-positive events leading to increased %PIT, particularly in normal control samples. In the assessment of Network 1, only pits in a small number of cells were counted whereas large numbers of cells and pits were analysed with Network 2. Furthermore, Network 1 and the manual annotators assessed the exact same cells, whereas Network 2 was compared to manual counts of only a proportion of the cells counted by the network.

A second aim of the study was to evaluate the durability of RBCs fixed in paraformaldehyde. We found no significant changes in %PIT after storing samples for up to 24 months and under varying temperatures and light exposures. These results agree with those from older studies, that found no alterations in PIT counts or RBC morphology in samples stored for up to 3 months at 4 and 20°C (Holroyde et al., 1969; Pearson et al., 1985). Establishing that %PIT is not affected significantly over time or in samples stored at room temperature, makes the method well suited for batch analyses in clinical trial settings. Furthermore, this may be useful in settings where transport to a facility with DIC equipment is necessary.

Our automated analysis produced a range of parameters that, to the best of our knowledge, have not previously been studied in detail. These include the number of pits per pitted cell (maximum and average) and average pit size. All parameters were found to correlate positively with %PIT. This accords with observations by Holroyde and Gardner in 1970 (Holroyde and Gardner, 1970), who found that pits in normal erythrocytes were usually small and single, with no one cell having >5 pits. In contrast, pits in cells from splenectomised individuals were larger and found in greater numbers, with up to 20 per one cell. Our studies suggest that as splenic function decreases, both the average size and number of pits per cell tends to increase, and it is possible that these parameters may be clinically useful. Future studies examining these in more detail are needed to establish whether they have any clinical predictive value.

Because there is no simple and direct measurement, splenic function is rarely assessed in clinical or research settings. In SCA, splenic function tests are an important tool for clinical trial settings. In milder sickle cell genotypes where hyposplenia may not affect all children, implementing splenic function measurements such as automated PIT counts in routine clinical follow-up could be an important tool for monitoring the risk of infection. Accessible splenic function tests may also prove useful in other conditions that involve loss of splenic function, such as thalassaemia, membranopathies and coeliac disease.

In summary, we have shown that automated PIT counts, produced using deep neural network analysis, are broadly comparable to manual counts. Automating PIT counts makes the method less time consuming and results comparable across laboratories. Furthermore, this workflow allows for larger numbers of RBCs to be studied, adding value to the analysis. Although the need for specialised microscopy equipment continues to limit widespread use, we believe that automated PIT counts can earn a place alongside HJB flow cytometry, being particularly useful in clinical trial settings where batch analyses are preferable, and in settings where samples cannot be analysed locally. We are currently working towards a streamlined workflow that will allow us to handle larger amounts of data and limit the number of steps and programs involved in the analysis. Once the workflow has been optimised, it is our intention to make all algorithms publicly available.

## Data Availability

The raw data supporting the conclusions of this article will be made available by the authors, without undue reservation.
